# Structure-Activity Relationships of Dopamine Transporter Pharmacological Chaperones

**DOI:** 10.3389/fncel.2022.832536

**Published:** 2022-05-09

**Authors:** Charles Sutton, Erin Q. Williams, Hoomam Homsi, Pieter Beerepoot, Reza Nazari, Dong Han, Amy J. Ramsey, Deborah C. Mash, David E. Olson, Bruce Blough, Ali Salahpour

**Affiliations:** ^1^Department of Pharmacology and Toxicology, Faculty of Medicine, University of Toronto, Toronto, ON, Canada; ^2^Departments of Neurology and Molecular and Cellular Pharmacology, University of Miami Miller School of Medicine, Miami, FL, United States; ^3^Department of Chemistry, College of Letters and Science, University of California, Davis, Davis, CA, United States; ^4^Department of Biochemistry and Molecular Medicine, School of Medicine, University of California, Davis, Davis, CA, United States; ^5^Center for Neuroscience, University of California, Davis, Davis, CA, United States; ^6^Center for Drug Discovery, RTI International, North Carolina, NC, United States

**Keywords:** dopamine transporter (DAT), ibogaine, bupropion, structure activity relationship, SAR, pharmacological chaperones

## Abstract

Mutations in the dopamine transporter gene (SLC6A3) have been implicated in many human diseases. Among these is the infantile parkinsonism-dystonia known as Dopamine Transporter Deficiency Syndrome (DTDS). Afflicted individuals have minimal to no functional dopamine transporter protein. This is primarily due to retention of misfolded disease-causing dopamine transporter variants. This results in a variety of severe motor symptoms in patients and the disease ultimately leads to death in adolescence or young adulthood. Though no treatment is currently available, pharmacological chaperones targeting the dopamine transporter have been shown to rescue select DTDS disease-causing variants. Previous work has identified two DAT pharmacological chaperones with moderate potency and efficacy: bupropion and ibogaine. In this study, we carried out structure-activity relationships (SARs) for bupropion and ibogaine with the goal of identifying the chemical features required for pharmacological chaperone activity. Our results show that the isoquinuclidine substituent of ibogaine and its analogs is an important feature for pharmacological chaperone efficacy. For bupropion, the secondary amine group is essential for pharmacological chaperone activity. Lastly, we describe additional ibogaine and bupropion analogs with varying chemical modifications and variable pharmacological chaperone efficacies at the dopamine transporter. Our results contribute to the design and refinement of future dopamine transporter pharmacological chaperones with improved efficacies and potencies.

## Introduction

The dopamine transporter (DAT) is a member of the solute carrier 6 (SLC6) family (Bu et al., 2021). DAT is a neuronal plasma membrane protein responsible for controlling extracellular levels of dopamine in addition to maintaining dopamine stores by transporting dopamine back into neurons after its release (Efimova et al., 2016). This recycling of dopamine is essential for locomotion, motivation, learning, and reward (Wise, 2004).

Dysfunction in the DAT is implicated in disorders including autism (Hamilton et al., 2013), attention deficit/hyperactive disorder (ADHD) (Mazei-Robison et al., 2008), Parkinson’s disease (Hansen et al., 2014), and the newly characterized infantile Parkinson’s-dystonia known as dopamine transporter deficiency syndrome (DTDS) (Ng et al., 2014). DTDS is a hereditary disease resulting from autosomal recessive loss-of-function mutations in the DAT sequence (Kurian et al., 2009).

The severity of DTDS is related to the level of mature DAT expression at the plasma membrane (Ng et al., 2014). Patients with variants that result in low DAT expression in heterologous cells experience a milder clinical phenotype than patients with variants resulting in no functional DAT expression. For some DTDS variants, the DAT protein does not mature past the endoplasmic reticulum (ER) and is not trafficked to the plasma membrane (Kurian et al., 2011). However, ER-retained DAT does not necessarily indicate a dysfunctional protein (Sanders and Myers, 2004). The stringent ER quality control will mark partially or even fully functional proteins for degradation if they are misfolded (Morello et al., 2000a). Indeed nearly 30% of newly synthesized wild-type (WT) proteins are immediately degraded (Schubert et al., 2000). For some membrane proteins, folding efficacy is less than 50% (Petäjä-Repo et al., 2000). However, small selective molecules known as pharmacological chaperones provide a unique way to address this problem of stringent ER quality control (Lindquist and Kelly, 2011; Beerepoot et al., 2017). Chemical chaperones are not selective and can help the folding of a wide range of proteins. Pharmacological chaperones differ from chemical chaperones in that they selectively target and bind to proteins in order to increase the proportion of the target protein that is properly folded. In fact, pharmacological chaperones have been used as therapeutic tools, for example, to rescue the vasopressin 2 receptor (Morello et al., 2000b) and the cystic fibrosis transmembrane conductance regulator (Hanrahan et al., 2013).

In a previous study, it was shown that noribogaine, a metabolite of ibogaine, acts as a DAT pharmacological chaperone, capable of rescuing DTDS and misfolded DAT variants in both cell culture and *Drosophila melanogaster* models (Kasture et al., 2016; Asjad et al., 2017). A recent structure-activity relationship (SAR) study also identified a tropane-based ibogaine analog that was capable of rescuing several DAT variants (Bhat et al., 2021). This analog also restored DAT function to WT levels for one variant, G386R. In another study, ibogaine and the atypical DAT inhibitor bupropion were able to rescue expression and function of select DTDS-causing variants in cells (Beerepoot et al., 2016). In contrast, typical inhibitors such as cocaine do not act as pharmacological chaperones for DAT. Interestingly the compounds that display pharmacological chaperone activity on DAT function as atypical inhibitors by stabilizing an inward-facing or occluded conformation of the transporter, as opposed to the outward-facing conformation induced by typical inhibitors (Bulling et al., 2012; Beerepoot et al., 2016).

Though the discovery of these DAT pharmacological chaperones is an important step to therapeutic intervention for DTDS, there are limitations with the current compounds that decrease their clinical utility. For instance, ibogaine, an efficacious chaperone, produces cardiotoxic effects at high concentrations (Koenig and Hilber, 2015). Bupropion, an effective antidepressant and smoking cessation intervention, has limited efficacy as a pharmacological chaperone. Both compounds have low potency (Beerepoot et al., 2016). Therefore, additional studies are required to improve the efficacy and potency of bupropion and ibogaine. Here, we describe SAR studies to identify the key molecular features necessary for the pharmacological chaperoning effect of these compounds. Our results demonstrate that the isoquinuclidine group of ibogaine and the secondary amine of bupropion are essential to confer high pharmacological chaperone efficacy. These studies will facilitate further refinement and design of improved DAT pharmacological chaperones.

## Materials and Methods

### Drugs

Bupropion hydrochloride and 6-hydroxybupropion were obtained from Toronto Research Chemicals (Toronto, Canada), ibogaine from Ibogaworld, cocaine from Medisca (Montreal, Canada), β-PEA, pinoline, indole, tryptamine, ephedrine, frovatriptan, alprenolol, isoproterenol, evodiamine, and tyramine from Sigma (Oakville, Canada), fluoxetine and amphetamine were from Tocris Bioscience (Bristol, United Kingdom). PAL analogs, RTI analogs, and bicifadine were synthesized at RTI in the Blough laboratory as described previously (Carroll et al., 2009, 2011; Blough et al., 2011, 2014). Noribogaine (Batch No. 606950002) was from Deborah Mash, Ph.D. (Obach et al., 1998). Ibogamine was obtained from Specs (Zoetermeer, The Netherlands). Sodium phenylbutyrate was purchased from Enzo Life Sciences Inc. (Farmingdale, NY). Ibogaminalog, ibogainealog, noribogainalog, fluorogainalog, and tabernanthalog were synthesized in the lab of David E. Olson (Cameron et al., 2020).

### Constructs

The β-lactamase (βlac) sequence was cloned from the ampicillin resistance gene within the pcDNA3.1 plasmid (Lam et al., 2013), using *Spe*I and *Asc*I restriction sites. An HA epitope was added to the N terminus of the βLAC, yielding HA-βLAC (Lam et al., 2013). To insert HA-βLAC into the second extracellular loop of YFP-HA-DAT, Quikchange II site-directed mutagenesis (Agilent, Mississauga, Canada) was used to insert *Spe*I and *Asc*I restriction sites on either side of the HA sequence of YFP-HA-DAT. The HA sequence in YFP-HA-DAT was removed by cuts with the *Spe*I and *Asc*I restriction enzymes prior to ligation of the HA-βLAC sequence. The *Spe*I and *Asc*I sites were then removed using site-directed mutagenesis, yielding YFP-HA-βLAC-DAT (referred to as βLAC-DAT). The human β2-adrenergic receptor (β2AR) cDNA expression vector was a gift from Dr. Michel Bouvier. HA-βLAC construct was subsequently inserted into this vector via *Asc*I and *Not*I restriction sites. Ligation of the HA-βLAC construct and into the β2AR cDNA expression vector yielded HA-βLAC-β2AR. To study the effects of alterations in the DAT, single point mutations were introduced into YFP-HA-DAT using Quikchange II site-directed mutagenesis. The resulting DAT variant and lysine-590-alanine (K590A), were confirmed by DNA sequencing.

### Transfections and Generation of Stable Cell Lines

HEK293 cells were obtained from ATCC (Manassas, VA) and maintained in Dulbecco’s Modified Eagle’s Medium (DMEM) (Sigma) supplemented with 10% FBS (Sigma), 100 U/mL penicillin and 100 μg/mL streptomycin. Cells were kept in 5% atmospheric CO_2_ and 37°C. Cells expressing SS-HA- βLAC- β 2AR were further supplemented with 1 μg/mL puromycin. All cells were treated with plasmocyin (250 μg/mL) for 2 weeks to ensure they were not infected.

On day 1, cells (2 × 10^6^) were seeded on a 10 cm tissue culture plate and incubated for 24 h. On day 2, seeded cells were transfected with 1–2 μg of plasmid DNA and 3 μL of polyethylenimine (1 mg/mL) (Polyscience Inc., Warminster, PA) per μg of DNA. Cells were then incubated in DMEM and transfection agent for 24 h. On day 3, to create stable lines, the media was replaced with media containing the selection agent G418 (500 μg/mL) (Bioshop, Burlington, Canada). In 5–7 days, most cells died and the media was replaced with fresh G418 selection media. The cells remaining were expanded until the 10 cm plate was approximately half-confluent. Cells were seeded onto 96-well plates at a density of 5 cells/mL with the goal of obtaining 1 cell per well. Plates were then incubated for 1–2 weeks. Clonal cell lines were generated by choosing wells with only 1 visible colony. These colonies were expanded and protein expression was confirmed via western blot or the βlac surface expression assay.

### Immunoblotting

Cells (1 × 10^6^ cells/well) were seeded onto a 6-well plate and incubated for 24 h. Cells were then treated with drugs and incubated for 16 h. After the incubation period, cells were lysed in RIPA buffer supplemented with protease inhibitors (working concentrations: pepstatin A, 5 μg/mL; leupeptin, 10 μg/mL; aprotinin, 1.5 μg/mL; benzamidine, 0.1 μg/mL; PMSF, 0.1 mM) on ice. At 4°C, cell lysates were gently shaken for 15 min followed by centrifugation at 15,000 rpm for 15 min to pellet debris. The Pierce BCA protein assay kit (Thermo Fisher Scientific, Canada) was used to determine the protein concentration of the supernatant. To reach a concentration of 40 μg of protein per 20 μL, samples were diluted in fresh RIPA buffer with 12.5% Laemmli sample buffer and 2.5% β-Mercaptoethanol prior to being heated to 55°C. The protein was then loaded on a 7.5 or 10% SDS-PAGE gel and run at 100 V for approximately an hour or until the dye front reached the bottom of the gel. Next, protein was transferred to a PVDF membrane at 22 V for 16 h or 100 V for 1 h. Blots were blocked with LI-COR blocking buffer for 1 h and incubated for 1 h at room temperature with rabbit anti-GFP (1:1,000 dilution; Life Technologies, cat#A11122). After washing three times with TBST, blots were incubated with goat anti-rabbit IRDye-680RD secondary antibody (1:15,000; LI-COR, cat#926-68071). As a loading control, blots were incubated with mouse anti-GAPDH (1:5,000, Sigma-Aldrich, Canada, cat#G8795) prior to secondary antibody goat anti-mouse IRDye-800GR (1:5,000 dilution, Rockland, Limerick, PA, cat#610-132-007). All antibodies were diluted in LI-COR blocking buffer. Additionally, REVERT total protein staining (LI-COR, Lincoln, NE) was used as a loading control. Drug treatments did not have an effect on GAPDH levels as determined by comparing GAPDH to total protein staining. Mature, fully glycosylated DAT (mDAT = 110 kDa) and immature, ER-resident DAT (iDAT = 75 kDa) were visualized using the Odyssey Imaging System (LI-COR, Lincoln, NE) and Image Studio Version 5.2 software (Supplementary Figure 1).

### β-Lactamase Surface Expression Assay

The βlac surface expression assay was performed by following the method outlined in Lam et al. (2013) and Beerepoot et al. (2015, 2016). The assay substrate nitrocefin (BD biosciences) was dissolved in dimethyl sulfoxide (DMSO) at a concentration of 10 mM and frozen at −80°C. Immediately before use, it was thawed and diluted to a final concentration of 100 μM in phosphate buffered saline (PBS). Cells (1 × 10^5^/well) were seeded into a poly-D-lysine-coated 48-well plate and incubated for 24 h, prior to drug treatment. Drugs were added to individual wells at varying concentrations and incubated for 16 h. Subsequently, the media was aspirated, and the cells were washed twice with PBS. After removal of the final PBS wash, 200 μL of the nitrocefin solution was added to each well and the absorbance was immediately read. The EPOCH microplate spectrophotometer (BioTek) was used to measure absorbance (486 nm) for each well every minute for 30 min. The rate of reaction (slope of the curve) was used as the readout for the assay (Beerepoot et al., 2015). To determine suitable concentration ranges for each drug, they were dissolved in PBS or DMSO at the highest concentration possible. From here, the solutions were serial diluted 1/10 to yield 8 different concentrations. βLAC cells were then treated with the varying drug concentrations and cell viability was recorded using light microscopy. For additional measure, nitrocefin was added and the absorbance was read. For certain cases, Trypan Blue was used to quantitatively differentiate increased cell death from reduced cell division. Drug doses that resulted in significant cell death and zero or heavily impaired reaction rates were not used in further experiments. Five or six doses were generally used in subsequent dosing experiments, starting at the highest non-toxic concentration.

### Dopamine Uptake

Dopamine uptake inhibition was conducted using the Neurotransmitter Transporter Uptake Assay Kit (Molecular Devices, prod#R8173). Cells (1 × 10^5^/well) were seeded into 96-well plates and incubated for 24 h. The cells were subjected to overnight drug treatment. The following day, wells were washed and incubated for 30 min with a mixture of 1X Hank’s Balanced Salt Solution (HBSS) and 0.1% Bovine Serum Albumin (BSA) Buffer. Dopamine uptake inhibitors were added during the incubation period. Following the incubation, the fluorescent dye was added, and the plate was immediately transferred to the bottom-read fluorescence microplate reader (Spectramax M3, Molecular Devices) for kinetic read-mode. The assay reads the amount of fluorescent activity which increases as fluorescent dye molecules are transported into the cells by the DAT. The read-out for this assay is the slope which equals the fluorescent measurements/time, ultimately denoting the uptake rate.

### Data Analysis and Statistics

Data analysis was performed using GraphPad Prism (GraphPad Software, La Jolla, CA). Linear regression analysis was performed on βLAC time points to determine the slope, thus the rate of reaction, for comparison. Furthermore, the slope was also used to determine the degree of dopamine uptake in the dopamine uptake assays. Non-linear regression, fitting to the log(agonist) vs. response and log(inhibitor) vs. response model curve, was used to determine dose-response relationships of surface expression and uptake assays, respectively. One-way ANOVA with Dunnett’s *post hoc*-test was used for the β-lactamase assay and one-way ANOVA with Bonferroni correction was used for western blot analysis.

## Results

### Bupropion Analogs Differ in Their Ability to Increase Dopamine Transporter Surface Expression

**FIGURE 1:**
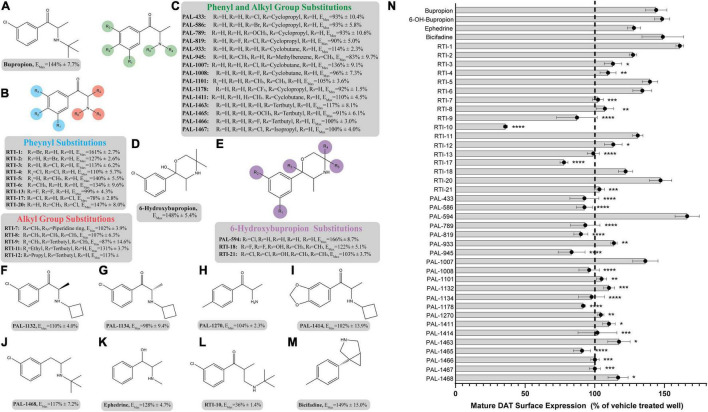
Structures of bupropion analogs and their maximal effects (E_Max_) on WT DAT surface expression. **(A)** Structure and E_Max_ of bupropion. **(B)** Bupropion analogs with phenyl substitutions (blue) or alkyl group substitutions (red). E_Max_ included for each compound. **(C)** Bupropion analogs with both phenyl and alkyl group substitutions. E_Max_ included for each compound. **(D)** Structure and E_Max_ of bupropion’s primary metabolite, 6-Hydroxybupropion. **(E)** 6-Hydroxybupropion analogs. E_Max_ included for each compound. **(F)** Structure and E_Max_ of bupropion analog PAL-1132. **(G)** Structure and E_Max_ of bupropion analog PAL-1134. **(H)** Structure and E_Max_ of bupropion analog PAL-1270. **(I)** Structure and E_Max_ of bupropion analog PAL-1414. **(J)** Structure and E_Max_ of bupropion analog PAL-1468. **(K)** Structure and E_Max_ of bupropion analog ephedrine. **(L)** Structure and E_Max_ of bupropion analog RTI-10. **(M)** Structure and E_Max_ of bupropion analog bicifadine. **(N)** Bar graph of bupropion analogs E_Max_ (****p < 0.0001, ***p < 0.001, **p < 0.01, and *p < 0.05, one-way ANOVA compared to bupropion with Dunnett’s test). Analogs are grouped in gray boxes based on similarities to the bupropion backbone. Data were normalized to vehicle treatment.

The pharmacological chaperone ability of 37 bupropion analogs, three 6-hydroxybupropion analogs, and the phenylamine uptake inhibitor bicifadine were measured using the microplate βlac surface expression assay (shown in Figure 1 and select compounds listed in Table 1). Importantly, this assay has been previously established and validated for measuring surface expression of DAT and was used by Lam et al. (2013) and Beerepoot et al. (2015) to show that bupropion and ibogaine are pharmacological chaperones of DAT. Figure 1 shows the results of HEK cells expressing YFP-HA-βlac-DAT that were incubated overnight with 100 μM concentrations of test drugs except for ephedrine which had a concentration of 1 mM. Previous experiments showed that bupropion increased WT DAT surface expression by 137% above vehicle treatment (Beerepoot et al., 2016) similar to what we are observing in this study, E_Max_ = 144 ± 7.7%. Many other bupropion analogs were also able to increase WT DAT surface expression. Analogs with minor derivations from the bupropion backbone had similar efficacy. Among these were RTI-1, RTI-2, RTI-5, RTI-6, RTI-11, RTI-20, and PAL-1007. Bupropion’s primary metabolite, 6-hydroxybupropion (148 ± 5.4%), and its analog, PAL-594 (166 ± 8.7%), also induced comparable surface expression effects as bupropion. Some compounds with larger derivations from the parent structure, such as ephedrine and bicifadine, were also able to increase WT DAT surface similar to bupropion. The E_Max_ values of all the analyzed drugs are included in Figure 1.

**TABLE 1:** Select bupropion analogs.

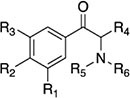			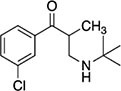		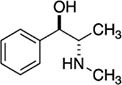		

							**Ephedrine**

**Compound**	**R1**	**R2**	**R3**	**R4**	**R5**	**R6**	**E_Max_ (%)**
Bupropion	Cl	H	H	CH_3_	H	tBu	144 ± 7.7%
**Phenyl substitution**							
RTI-1	Br	H	H	CH_3_	H	tBu	161 ± 2.7%
RTI-2	H	Br	H	CH_3_	H	tBu	127 ± 2.6%
RTI-3	H	Cl	H	CH_3_	H	tBu	113 ± 6.2%
RTI-4	Cl	Cl	H	CH_3_	H	tBu	110 ± 5.7%
RTI-5	H	CH_3_	H	CH_3_	H	tBu	140 ± 5.5%
RTI-6	CH_3_	H	H	CH_3_	H	tBu	134 ± 9.6%
RTI-13	F	F	H	CH_3_	H	tBu	99 ± 4.3%
RTI-17	Cl	H	Cl	CH_3_	H	tBu	78 ± 2.8%
RTI-20	Cl	CH_3_	H	CH_3_	H	tBu	147 ± 8.0%
**Alkyl group substitution**							
RTI-7	Cl	H	H	CH_3_		Piperidine	102 ± 3.9%
RTI-8	Cl	H	H	CH_3_	CH_3_	CH_3_	107 ± 6.3%
RTI-9	Cl	H	H	CH_3_	CH_3_	tBu	87 ± 14.6%
PAL-1007	Cl	H	H	CH_3_	H	Cyclobutane	136 ± 9.1%
RTI-11	Cl	H	H	CH_2_CH_3_	H	tBu	131 ± 3.7%
RTI-12	Cl	H	H	CH_2_CH_2_CH_3_	H	tBu	113 ± 6.3%
PAL-1270	H	CH_3_	H	CH_3_	H	H	104 ± 2.3%
**Other analogs**							
Ephedrine	−	−	−	−	−	−	128 ± 4.7%
RTI-10	−	−	−	−	−	−	36 ± 1.4%
PAL-945	H	CH_3_	H	CH_3_	CH_3_	Benzyl	83 ± 9.7%
PAL-1101	CH_3_	H	H	CH_3_	H	CH_3_	105 ± 3.6%
PAL-1411	CH_3_	H	H	CH_3_	H	Cyclobutane	110 ± 4.5%

**FIGURE 2:**
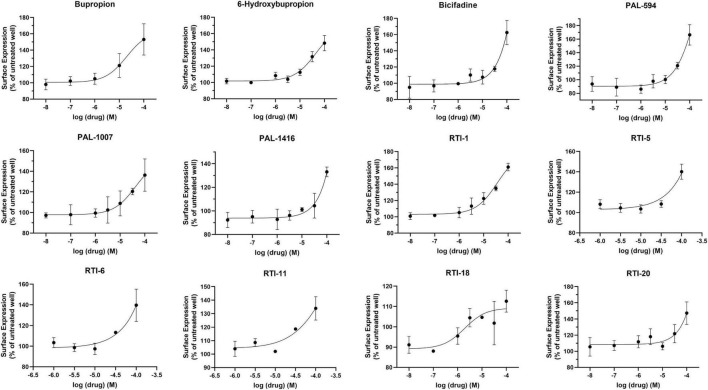
Effect of overnight bupropion analog treatment on YFP-HA-βlac-DAT surface expression. Dose-dependent curves for most hit compounds of the bupropion backbone. Data were normalized to vehicle treatment. Data were fitted to one-site dose-response non-linear regression curves using GraphPad Prism. Data are means ± S.E.M. *n* = 3–6.

Dose-response curves were generated to assess the potency of compounds in Figure 1. None of the tested compounds in Figure 1 displayed an EC_50_ that was significantly better than bupropion (data not shown). Dose response curves for select compounds with varying chemical modifications of the bupropion backbone are shown in Figure 2. For most compounds, full dose-response curves were not obtained since higher concentrations of drugs induced cellular toxicity. As such the values reported in this study are assumed effective E_Max_. Of note, the chaperone potency of bupropion in the βlac assay (39 μM) is nearly 100-fold higher and right-shifted compared to the reported IC_50_ value of bupropion for dopamine uptake inhibition (0.6 μM) (Carroll et al., 2010). Therefore we next determined whether the insertion of HA-βlac into the second extracellular loop of DAT resulted in a reduction of bupropion’s uptake inhibition potency compared to YFP-HA-DAT (WT DAT). Our results show that within our experimental conditions, bupropion’s IC_50_ for inhibiting uptake activity of YFP-HA-βlac is 1.3 μM, similar to that of the WT construct (0.84 μM) (Supplementary Figure 2).

### Ibogaine and Tryptamine Analogs Differ in Their Ability to Increase Dopamine Transporter Surface Expression

**FIGURE 3:**
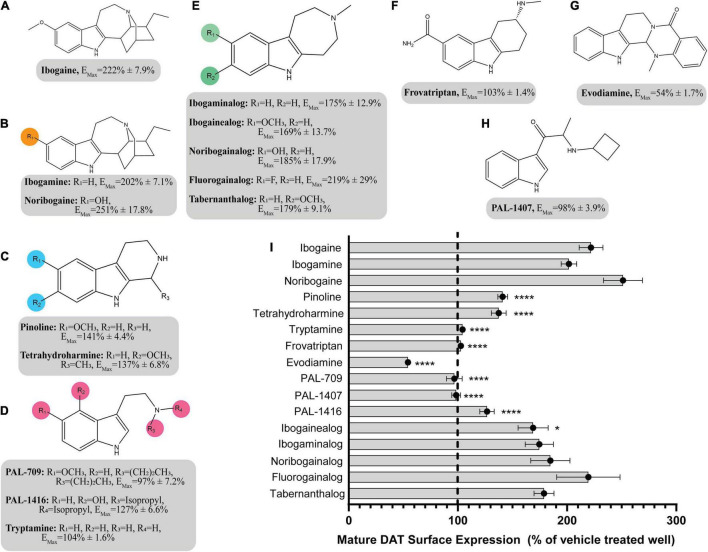
Structures of ibogaine analogs and their effects (E_Max_) on WT DAT surface expression. **(A)** Structure and E_Max_ of ibogaine. **(B)** Ibogaine analogs. E_Max_ included for each compound. **(C)** The βCarbolines. E_Max_ included for each compound. **(D)** Tryptamine analogs. E_Max_ included for each compound. **(E)** Analogs without the isoquinuclidine substituent. E_Max_ included for each compound. **(F)** Structure and E_Max_ of frovatriptan. **(G)** Structure and E_Max_ of evodiamine. **(H)** Structure and E_Max_ of PAL-1407. **(I)** Bar graph of ibogaine analogs E_Max_ (****p < 0.0001 and *p < 0.05, one-way ANOVA compared to ibogaine with Dunnett’s test). Analogs are grouped in gray boxes based on similarities to the ibogaine backbone. Data were normalized to vehicle treatment.

**FIGURE 4:**
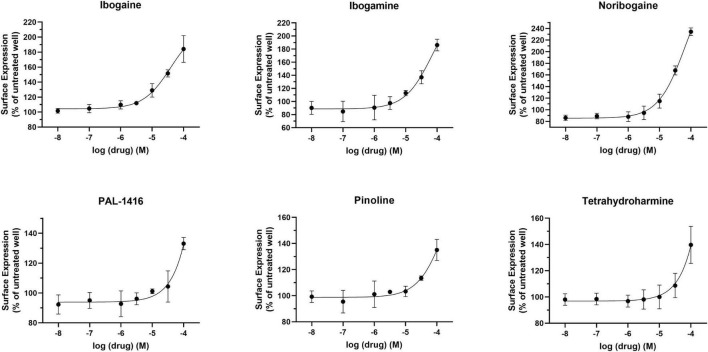
Effect of overnight ibogaine analog treatment on YFP-HA-βlac-DAT surface expression. Dose-dependent curves for ibogaine analogs of interest. Data were normalized to vehicle treatment. Data were fitted to one-site dose-response non-linear regression curves using GraphPad Prism. Data are means ± S.E.M. *n* = 3–6.

The pharmacological chaperone ability of the 15 ibogaine and tryptamine analogs, shown in Figure 3, were measured using the βlac surface expression assay. HEK cells expressing YFP-HA-βlac-DAT were incubated overnight with these compounds at concentrations varying by log and half-log dilutions, starting at 100 μM final concentration. Here, it was of particular interest to ascertain whether any of the ibogaine analogs could display a greater E_Max_ and/or more potent EC_50_ than what was observed with the bupropion analogs. Furthermore, it was important to determine whether the β-carboline analogs could also elicit a chaperone effect because they are not classified as DAT ligands (Cao et al., 2007). Our results here showed that ibogaine has an E_Max_ of 222 ± 7.9%, close to what we reported previously (187%) (Beerepoot et al., 2016). Analogs ibogamine and noribogaine were also shown to have high efficacy for increasing WT DAT surface expression. Ibogaine analogs lacking the isoquinuclidine substituent had varied efficacy. One compound, ibogainealog (169 ± 13.7%) had less efficacy than ibogaine (222 ± 7.9%). The βcarbolines pinoline (141 ± 4.4%) and tetrahydroharmine (137 ± 6.8%) also had reduced efficacy compared to ibogaine. Removing the nitrogen from the ring reduced efficacy as observed with frovatriptan (103 ± 1.4%). Tryptamine analogs were significantly less efficacious than ibogaine. Evodiamine (54 ± 1.7%) had no chaperone ability and caused a substantial reduction in the surface expression of DAT. Dose-response curves were generated to assess the pharmacological chaperone potencies of select compounds (Figure 4). As with the bupropion analogs, there were only modest differences in EC_50_ values of the ibogaine and tryptamine analogs.

### Test Compounds Are Selective Pharmacological Chaperones of Dopamine Transporter

To ascertain whether the increase in DAT surface expression shown in Figures 1, 3 were the result of selective pharmacological chaperone activity on the dopamine transporter, we tested the effects of select compounds on the surface expression of β2AR, an unrelated membrane protein. Alprenolol and isoproterenol were used as positive controls for increasing or decreasing β2AR surface expression, respectively (January et al., 1997; Kobayashi et al., 2009; Lam, 2013). The tested bupropion and ibogaine analogs did not significantly increase β2AR surface expression, demonstrating that their effects are selective for DAT (Supplementary Figures 3A,B).

### Dopamine Transporter Pharmacological Chaperones Increase Total Dopamine Transporter Protein Levels

We next assessed whether drugs that increase DAT surface expression also increase total protein levels of DAT. Cells expressing YFP-HA-DAT were incubated with select test compounds and DAT protein levels were examined by western blot. The effects of the compounds were assessed on WT DAT and K590A DAT. The K590A mutant is a well-characterized ER-retained, trafficking-deficient DAT mutant (Miranda et al., 2004) that can be rescued by pharmacological chaperones (Beerepoot et al., 2016).

**FIGURE 5:**
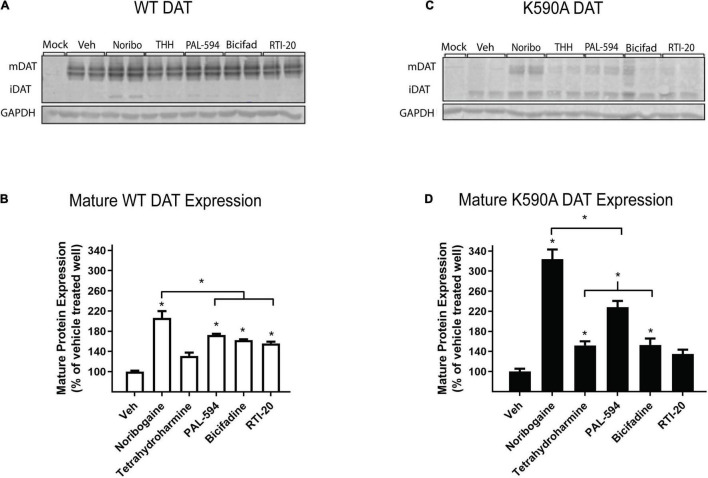
Effect of noribogaine, tetrahydroharmine, PAL-594, bicifadine, and RTI-20 on WT and K590A DAT expression. **(A)** Representative blot of WT DAT cells treated overnight with 100 μM noribogaine, tetrahydroharmine, PAL-594, Bicifadine, and RTI-20. Mature, fully glycosylated DAT (mDAT = 110 kDa) and immature, ER-resident DAT (iDAT = 75 kDa) are shown. **(B)** Quantification of WT mDAT protein levels. Data are means ± S.E.M; * = *P* < 0.05; *n* = 3–4. **(C)** Representative blot of K590A DAT cells. Mature, fully glycosylated DAT (mDAT = 110 kDa) and immature, ER-resident DAT (iDAT = 75 kDa) are shown. **(D)** Quantification of K590A mDAT protein levels. Data are means ± S.E.M; * = *P* < 0.05; *n* = 3–4.

We compared the effects of the following five compounds on DAT protein expression: noribogaine (251 ± 17.8%), tetrahydroharmine (137 ± 6.8%), PAL-594 (166 ± 8.7%), bicifadine (149 ± 15.0%), and RTI-20 (147 ± 8.0%). These compounds were chosen because they have varying efficacy in increasing DAT surface expression. As shown in Figure 5, all compounds, with the exception of tetrahydroharmine, were able to significantly increase mature WT DAT protein levels, with noribogaine having the largest effect (Figures 5A,B). Similarly all compounds except RTI-20 had a statistically significant effect on K590A protein levels (Figures 5C,D). Overall, these data suggest that noribogaine and PAL-594 are the most efficacious chaperones for both WT and K590A DAT as assessed by western blot and the βlac surface expression assay.

### Dopamine Transporter Inhibition and Dopamine Transporter Chaperoning Are Distinct Processes

When assessing the pharmacological chaperone efficacies of the test compounds, we noted that the reported DAT binding affinity and dopamine uptake inhibition values of the studied analogs did not appear to be predictive of their chaperone ability. These results are summarized in Supplementary Table 1, along with DAT binding and dopamine uptake inhibition data from Carroll et al. (2009, 2010, 2014). Five compounds, bupropion (144 ± 7.7%), RTI-2 (127 ± 2.6%), RTI-5 (140 ± 5.5%), RTI-6 (134 ± 9.6%), and RTI-11 (131 ± 3.7%), highlight the major trends in the table. Specifically, RTI-5 and RTI-11 show similar surface expression chaperoning efficacies of 140 ± 5.5 and 131 ± 3.7%, respectively. However, RTI-5 (140 ± 5.5%) has a K_i_ of > 10,000 nM and an IC_50_ of 6,840 nM, thus suggesting it has poor inhibition and essentially no binding affinity for DAT (Carroll et al., 2009). Comparatively, RTI-11 (131 ± 3.7%) has a K_i_ of 459 nM and IC_50_ of 31 nM (Carroll et al., 2014). RTI-4 (110 ± 5.7%) has nearly no chaperone effect on DAT but is reported to bind strongly to DAT and is an effective inhibitor of dopamine uptake (K_i_ = 472 nM and IC_50_ = 271 nM) (Carroll et al., 2009). These data suggest that there is low correlation between pharmacological chaperone efficacy and classical DAT binding affinity or dopamine uptake inhibition of DAT compounds.

**FIGURE 6:**
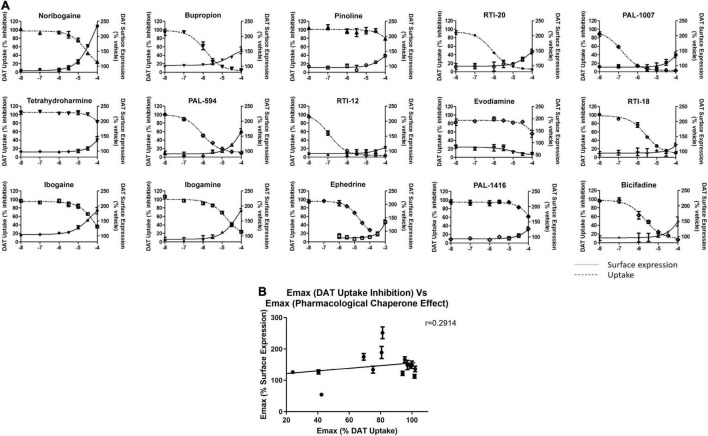
Comparison of dopamine uptake inhibition and DAT surface expression for hit compounds. **(A)** Dose responses of 15 hit compounds are represented with uptake inhibition represented on the left *Y*-axis and surface expression on the right *Y*-axis. Doses used are half-log dilutions between 100 μM and 100 nM, with 2 full log dilutions yielding 10 and 1 nM. Data were normalized to vehicle treatment. Data were fitted to one-site dose-response non-linear regression curves using GraphPad Prism. Data are means ± S.E.M. **(B)** Correlation graph of pharmacological chaperone E_Max_ (*y*-axis) and dopamine uptake inhibition E_Max_ (*x*-axis) of the 15 compounds in **(A)**. There was low correlation between pharmacological chaperone effect and dopamine uptake inhibition as demonstrated by the Pearson correlation coefficient, *r* = 0.2914. Data are means ± S.E.M.

Following this observation, we tested select chaperone compounds to see if they followed the same trend. Figure 6A shows dose response curves of uptake inhibition (left *Y*-axis) and surface expression chaperoning efficacy (right *Y*-axis) of select test compounds. This figure also shows that a compound’s inhibitory effect on dopamine uptake is not strongly correlated with its chaperoning efficacy. Two curves that clearly show the absence of a strong correlation are noribogaine (251 ± 17.8%) and RTI-12 (113 ± 6.3%). Noribogaine has a maximal dopamine uptake inhibition of 76%, whereas RTI-12 has a maximal inhibition of 96%. Interestingly, the effects are reversed with regards to pharmacological chaperoning. Figure 6B shows that indeed there is low correlation relationship between uptake inhibition and chaperone efficacy. Altogether, the results in Figure 6 indicate that pharmacological chaperone and inhibitory effects of DAT compounds are distinct processes.

## Discussion

### Structure-Activity Relationship: Bupropion Scaffold

Based on the study of 37 bupropion analogs, certain structural features of bupropion appear to be necessary to increase WT DAT surface expression. Phenyl substitution seemed to be an important factor in chaperone activity. The para-bromo compound RTI-2 (127 ± 2.6%) has an efficacy similar to bupropion while the para-chloro compound RTI-3 (113 ± 6.2%) is less efficient than bupropion. Replacing a chlorine with a methyl group also affects chaperone activity, as observed when replacing the chlorine in PAL-1007 (136 ± 9.1%), which has a similar efficacy to bupropion, with a methyl in PAL-1411 (110 ± 4.5%), which is less efficient than bupropion. A similar trend is observed when comparing bupropion (144 ± 7.7%) with RTI-6 (134 ± 9.6%) although not statistically significant (Supplementary Figure 4B). Meta-substitution leads to more efficaciousness than para-substitution. This is observed when comparing bupropion (144 ± 7.7%) to RTI-3 (113 ± 6.2%) and comparing RTI-1 (161 ± 2.7%) to RTI-2 (127 ± 2.6%) which shows a similar trend (Supplementary Figure 4A). The addition of a second chloride to the ring caused a dramatic reduction in efficacy, essentially eliminating chaperone activity, as seen in RTI-4 (110 ± 5.7%), RTI-17 (78 ± 2.8%), and RTI-21 (103 ± 3.7%) (Supplementary Figure 4C). Interestingly, the addition of the second halogen has a favorable effect on DAT binding affinity and dopamine uptake (Supplementary Table 1) but is deleterious for chaperone efficacy.

A broader study of 3-phenyl substituents on bupropion showed that no other substitution improved chaperone activity. In fact, most substitutions rendered the compound inactive and less efficacious than bupropion as seen with 3-methoxy compound PAL-1465 (91% ± 6.1) and 3-fluoro compound PAL-1466 (100% ± 3.0%) (Supplementary Figure 5A). Similar sets of compounds were studied with an N-cyclopropyl and N-cyclobutyl in place of the N-tertbutyl group and a similar trend was observed. The N-cyclobutyl series mirrored the N-terbutyl series, with the 3-chloro compound PAL-1007 (136 ± 9.1%) being similar to bupropion, while the unsubstituted analog PAL-993 (114 ± 2.3%) and 3-methyl analog PAL-1411 (110 ± 4.5%) were less efficacious than bupropion (Supplementary Figure 5B). The 3-fluoro analog PAL-1008 (96 ± 7.3%) was inactive. A series of N-cyclopropyl compounds was also synthesized but surprisingly, none of the compounds were active (Supplementary Figure 5C). PAL-433 (93 ± 10.4%), PAL-586 (93 ± 5.8%), PAL-789 (93 ± 10.6%), PAL-819 (90 ± 5.0%), and PAL-1178 (92 ± 1.2%) were all inactive.

The cyclopropyl results suggest that N-alkylation is an important factor in chaperone activity, one that possibly overrides the effects of phenyl substitution. Tertiary amines RTI-7 (102 ± 3.9%), RTI-8 (107 ± 6.3%), RTI-9 (87 ± 14.6%), and PAL-945 (83 ± 9.7%) were less effective than bupropion (Supplementary Figure 6A). Smaller alkyl groups such as in the N-methyl compound PAL-1101 (105 ± 3.6%), and the primary amine PAL-1270 (104 ± 2.3%) were also less effective than bupropion (Supplementary Figure 6B). Interestingly, the N-cyclobutyl analog PAL-1007 (136 ± 9.1%), had similar activity to bupropion but the N-cyclopropyl analog PAL-433 (93 ± 10.4%) was completely inactive. The N-isopropyl analog PAL-1467 (100 ± 4.0%) was also inactive (Supplementary Figure 6C). These data suggest that chaperone activity required a somewhat bulky alkyl group on the amine, but a hydrogen was also needed. These trends may correlate with basicity or possibly, lipophilicity.

Differences in the alkyl chain as well as lengthening the link between the amine and phenyl ring also affected chaperone efficacy. Lengthening the chain appeared to have a negative effect on chaperone efficacy (Supplementary Figure 7A). Bupropion (144 ± 7.7%) and RTI-12 (113 ± 6.3%) have methyl and propyl groups, respectively. The addition of a methylene group in RTI-10 (36 ± 1.4%) also eliminated the chaperone effect, and in fact may have induced DAT internalization (Supplementary Figure 7B). We also observed that a ketone is not required for chaperone efficacy, as seen with ephedrine (128 ± 4.7%) (Supplementary Figure 7C).

Interestingly, the primary human metabolite of bupropion, 6-hydroxybupropion (148 ± 5.4%), which is obtained by oxidation of one of the t-butyl methyl groups, had similar efficacy as bupropion (144 ± 7.7%). This is a bit surprising given the large structural differences between the two scaffolds. However, phenyl morpholines have been shown to be good DAT ligands (Lukas et al., 2010). As with the bupropion analogs, the di-chloro 6-hydroxybupropion analog RTI-21 (103 ± 3.7%) was less efficacious than bupropion (Supplementary Figure 8A). PAL-594 (166 ± 8.7%), in which the hydroxyl and methyl groups have been removed had similar efficacy as bupropion (Supplementary Figure 8B). This was an interesting finding because one explanation for the activity of 6-hydroxybupropion could be that the compound binds in its ring-opened form, which is bupropion with a hydroxyl on one of the t-butyl methyl groups (Supplementary Figure 9). PAL-594 (166 ± 8.7%) cannot ring open suggesting that its activity is probably due to the ring-closed morpholine structure. This should be explored further to elucidate the mechanism behind this observation. Finally, the small phenylamine triple uptake inhibitor bicifadine (149 ± 15.0%) (Basile et al., 2007) was found to be just as efficacious as bupropion and may represent another scaffold from which to develop pharmacological chaperones (Supplementary Figure 10A). It should be noted that the 4-methyl analog of bupropion RTI-5 (140 ± 5.5%) was also similarly efficacious as bupropion (Supplementary Figure 10B).

### Structure-Activity Relationship: Ibogaine Scaffold

The SAR of the ibogaine backbone was not nearly as extensive as that of bupropion due to the difficulty of synthesizing ibogaine analogs. However, several structural trends were evident. Although not statistically significant, our observations show that replacing the methoxy group of ibogaine (222 ± 7.9%) with a hydroxyl group on noribogaine (251 ± 17.8%) results in a slightly higher chaperone efficacy trend. Interestingly, removal of the methoxy group resulted in trends toward reduction of chaperone efficacy, as seen with ibogamine (202 ± 7.1%) (Supplementary Figure 11). Our observatons are in line with reports from other groups showing that noribogaine has a slightly bigger efficacy than ibogaine as a pharmacological chaperone (Kasture et al., 2016).

Deconstruction of the ibogaine ring system was explored in order to determine if the complex ring system is required for chaperone activity. Three of the seven analogs with the isoquinuclidine ring removed were less efficacious than ibogaine (pinoline, tetrahydroharmine, and ibogainealog). The methoxy, hydroxy, and unsubstituted analogs ibogainealog (169 ± 13.7%), ibogaminalog (175 ± 12.9%), noribogainalog (185 ± 17.9%), and tabernanthalog (179 ± 9.1%) lost approximately half their chaperone efficacy compared to ibogaine (222 ± 7.9%) with only ibogainealog achieving statistical significance (Figure 3 and Supplementary Figure 12A). The fluorinated compound, fluorogainalog (219 ± 29.0%), had an efficacy comparable to ibogaine (222 ± 7.9%). Interestingly, a recent paper by Bhat et al. (2021), also reported that a fluorinated tropane analog of ibogaine had enhanced chaperone efficacy on DAT. In fact, in our studies we also observed that the presence of other halogens such as bromine and chlorine were shown to be toxic for cells (data not shown). The tetrahydroharmine pinoline (141 ± 4.4%) which has lower efficacy than ibogaine, lacks the isoquinuclidine ring and contains a smaller ring (6-membered vs. 7-membered), suggesting that ring size matters (Supplementary Figure 12B). The natural product evodiamine (54 ± 1.7%), which has a tetrahydroharmine embedded in the structure, was also studied. Surprisingly, evodiamine may also induce DAT internalization, with an E_Max_ of only 54%.

Tryptamines are biogenic amine compounds with activity on transporters but generally, they do not have chaperone efficacy in the βlac assay (Supplementary Figure 13). Tryptamine (104 ± 1.6%), PAL-709 (97 ± 7.2%), PAL-1416 (127 ± 6.6%), and PAL-1407 (98 ± 3.9%) vary by ring substitution and N-alkylation and were all inactive or had very little chaperone activity relative to ibogaine (222 ± 7.9%). Tryptamines were studied because they are more deconstructed analogs of ibogaine and it is possible they could adapt a suitable conformation to be chaperones, similar to noribogaine (251 ± 17.8%), due to the highly flexible carbon chain and tertiary amine. However, their E_Max_ values appear to indicate otherwise and thus, further work on the tryptamine scaffold may be futile.

When combined, these studies show that the complex ring system of ibogaine may be required for chaperone activity. In support of this, a recent study has shown that several ibogaine analogs, with the isoquinuclidine ring replaced by a tropane ring, maintain similar levels of chaperone efficacy as ibogaine (Bhat et al., 2021). In that study one or two analogs also displayed enhanced potency.

### Discrepancy Between Dopamine Uptake Inhibition and Chaperoning Effect

Figure 6 shows there is very low correlation between pharmacological chaperone efficacy and dopamine uptake inhibition. Conventionally, this discrepancy could be explained by differing binding affinities between the extracellular transporter and the ER-resident intermediates. Indeed, it is possible that changes in intracellular ion concentrations could alter binding affinity of inhibitors (Amejdki-Chab et al., 1992; Chen et al., 2002). Furthermore, the immature DAT intermediates that are the targets for pharmacological chaperones are likely to have different affinities for inhibitors due to incomplete folding, unprocessed glycosylation, and interactions with chaperone proteins. Generally, pharmacological chaperones have lower potencies than what their reported binding affinities would suggest (Leidenheimer and Ryder, 2014). Though this explanation could provide insight to the observed dosing discrepancy, it does not explain the lack of correlation between the two effects.

One other explanation is that the various ligands stabilize different conformations of DAT. It is reported that classical inhibitors, such as cocaine, stabilize an outward-facing conformation of the transporter while atypical inhibitors, such as bupropion and ibogaine, induce an inward-facing or occluded conformation (Schmitt et al., 2013). Previous studies have suggested that compounds inducing an inward-facing or occluded conformation also exhibit a chaperone effect, as is the case with bupropion and ibogaine (Beerepoot et al., 2016). As such, some of the discrepancies we have noted with the analogs tested in this study could potentially be due to the different conformations stabilized by these compounds. Therefore, greater chaperoning efficacy may indicate that the analogs stabilize inward-facing or occluded DAT conformations. Future molecular dynamics and modeling studies will be needed to better understand what conformations within the transporter underlie the pharmacological chaperone activity of various compounds.

## Conclusion

In this study we identified some key structural features required for high chaperone efficacy on DAT (Supplementary Figure 14). These include the isoquinuclidine group and hydroxyl substituent on the ibogaine backbone. For bupropion we found that the secondary amine and halogen derivation at the meta position are necessary for chaperone activity. While we did identify these important features that affect chaperone efficacy, we were less successful in identifying features that would increase potency. Thus, future work should be focused on identifying compounds with increased chaperone potency. A recent study identified a tropane-based ibogaine analog that displayed high efficacy and restored dopamine uptake of a DTDS disease-causing DAT variant up to 40% of that of WT DAT. This exceeds the restored uptake by noribogaine by approximately fourfold (Bhat et al., 2021). Studies such as these lay the groundwork for discovering more potent DAT pharmacological chaperones. By consolidating SAR data from our study and others, the search for potent and efficacious DAT pharmacological chaperones will be greatly accelerated.

## Data Availability Statement

The original contributions presented in the study are included in the article/Supplementary Material, further inquiries can be directed to the corresponding author/s.

## Author Contributions

CS, EQW, HH, PB, RN, and DH carried out the experiments. CS, EQW, HH, PB, RN, DH, AJR, BB, and AS analyzed data. EQW, BB, and AS wrote the manuscript. CS, EQW, HH, PB, RN, DH, AJR, DCM, DEO, BB, and AS edited the manuscript. DCM, DEO, and BB contributed unique reagents. All authors contributed to the article and approved the submitted version.

## Conflict of Interest

DEO is a co-founder of Delix Therapeutics, Inc., and currently serves as the Chief Innovation Officer and Head of the Scientific Advisory Board. DCM is an inventor on patents pertaining to noribogaine. She is the CEO, founder and a shareholder in DemeRx, Inc. The remaining authors declare that the research was conducted in the absence of any commercial or financial relationships that could be construed as a potential conflict of interest.

## Publisher’s Note

All claims expressed in this article are solely those of the authors and do not necessarily represent those of their affiliated organizations, or those of the publisher, the editors and the reviewers. Any product that may be evaluated in this article, or claim that may be made by its manufacturer, is not guaranteed or endorsed by the publisher.

## Supplementary Material

Supplementary Figure 1 YFP-HA-DAT Immunoblot Bands. First lane: molecular weight ladder. Second lane: YFP-HA-DAT with two bands representing mature DAT (110 kDa) and immature, ER-resident DAT (75 kDa).

Supplementary Figure 2 Dose response inhibition of dopamine uptake by bupropion on WT and YFP-HA-βlac-DAT. Data were normalized to vehicle treatment. Data are means ± S.E.M.

Supplementary Figure 3 E_Max_ comparison between βlac-DAT and βlac-β2AR surface expression of hit compounds showing selectivity of chaperones for βlac-DAT. **(A)** Summary of the maximum observed effects of hit compounds on WT DAT surface expression. Data were normalized to vehicle treatment and analyzed with one-way ANOVA (*****p* < 0.0001, ****p* < 0.001, ***p* < 0.01, and **p* < 0.05, one-way ANOVA compared to vehicle with Dunnett’s test). **(B)** Summary of the maximum observed effects of hit compounds on β2AR. Data were normalized to vehicle treatment and analyzed with one-way ANOVA (*****p* < 0.0001, ****p* < 0.001, ***p* < 0.01, and **p* < 0.05, one-way ANOVA compared to vehicle with Dunnett’s test). The final concentration of drugs used was 100 μM, except for ephedrine at 1 mM. Data are means ± S.E.M; *n* = 3–6.

Supplementary Figure 4 Impact of phenyl substitutions on the efficacy of bupropion in the WT DAT blac assay. **(A)** Impact of para substitution compared to meta substitution on chaperone efficacy. **(B)** Impact of phenyl substitution at the meta position on chaperone efficacy. **(C)** Impact of addition of a second Cl on chaperone efficacy.

Supplementary Figure 5 Broader study of the impact of phenyl substitutions on the efficacy of bupropion in the WT DAT blac assay. **(A)** Impact of 3-phenyl substitutions on chaperone efficacy of compounds with an N-tertbutyl group. **(B)** Impact of 3-phenyl substitutions on chaperone efficacy of compounds with an N-cyclobutyl group. **(C)** Impact of 3-phenyl substitutions on chaperone efficacy of compounds with an N-cyclopropyl group.

Supplementary Figure 6 Impact of N-alkylation on the efficacy of bupropion in the WT DAT blac assay. **(A)** Impact of tertiary amines on chaperone efficacy. **(B)** Impact of smaller alkyl groups on chaperone efficacy. **(C)** Impact of iso-alkyl groups on chaperone efficacy.

Supplementary Figure 7 Effect of the alkyl chain length and ketone substituent on the efficacy of bupropion in the WT DAT blac assay. **(A)** Impact of alkyl chain length on chaperone efficacy. **(B)** Impact of the addition of a methylene group on chaperone efficacy. **(C)** Impact of the addition of a ketone on chaperone efficacy.

Supplementary Figure 8 Efficacy of 6-hydroxybupropion in the WT DAT blac assay.**(A)** Impact of the addition of a second halogen on chaperone efficacy of 6-hydroxybupropion. **(B)** Impact of the removal of hydroxyl and methyl groups from 6-hydroxybupropion on chaperone efficacy.

Supplementary Figure 9 Demonstration of bupropion converted to its metabolite 6-hydroxybupropion.

Supplementary Figure 10 Bicifadine and RTI-5 are potential bupropion analogues to be explored further for SAR analysis. **(A)** Bupropion and bicifadine have similar chaperone efficacy. **(B)** Bupropion and RTI-5 have similar chaperone efficacy.

Supplementary Figure 11 Effect of the hydroxyl substitution on the efficacy of ibogaine in the WT DAT βlac assay.

Supplementary Figure 12 Effect of the isoquinuclidine substituent and ring size on the efficacy of ibogaine in the WT DAT blac assay. **(A)** Impact of the removal of the isoquinuclidine substituent in addition to various phenyl substitutions on chaperone efficacy. **(B)** Impact of ring size on chaperone efficacy.

Supplementary Figure 13 Effect of the tryptamines on the efficacy of tryptamine analogs in the WT DAT βlac assay.

Supplementary Figure 14 Key structural features of bupropion and ibogaine for pharmacological chaperone efficaciousness. **(A)** Bupropion’s key structural features for pharmacological chaperone activity include the halogen derivation at the meta position (pink) and the secondary amine (green). **(B)** Ibogaine’s key structural features for pharmacological chaperone activity include hydroxyl substitution (blue) and the presence of the isoquinuclidine group (orange).
