# Supplementary material for: Structure-Activity Relationships of Dopamine Transporter Pharmacological Chaperones
Source: Front Cell Neurosci. 2022 May 9;16:832536. doi: 10.3389/fncel.2022.832536 (PMC9124866; doi:10.3389/fncel.2022.832536)
Supplement: Supplementary file 1 [file Table_1.docx]

**Supplemental Table 1: Comparison of surface expression E_max_, DAT binding, and dopamine uptake inhibition values for bupropion and RTI 2-11 in WT DAT cells. DAT binding and dopamine uptake inhibition values from [26][32][33].**
