# Supplementary figures and images for: Structure-Activity Relationships of Dopamine Transporter Pharmacological Chaperones
Source: Front Cell Neurosci. 2022 May 9;16:832536. doi: 10.3389/fncel.2022.832536 (PMC9124866; doi:10.3389/fncel.2022.832536)

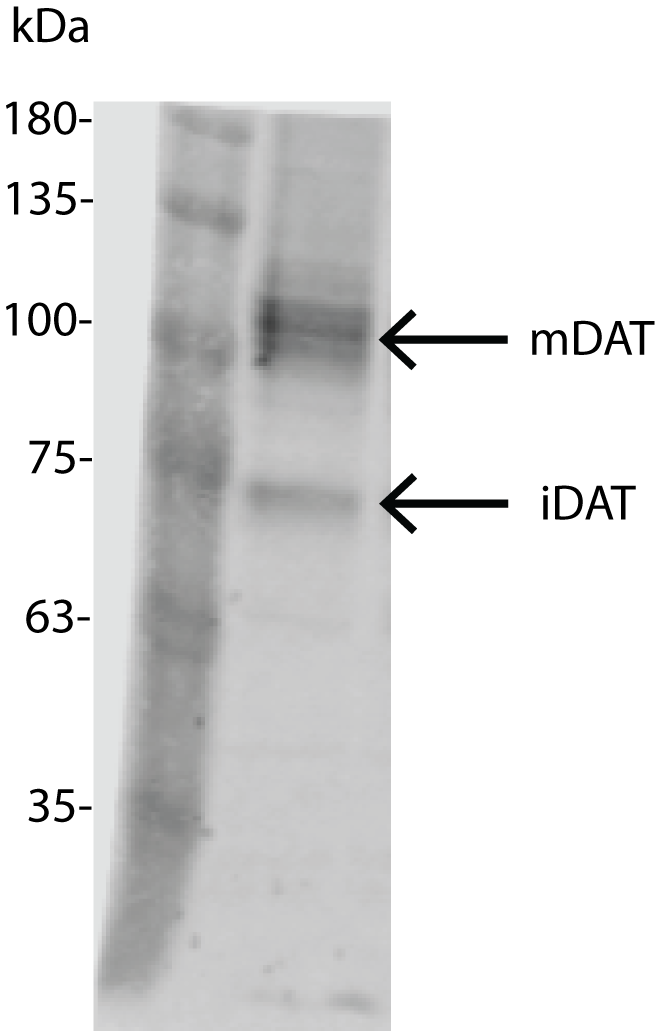

Supplement: Supplementary Figure 1 — YFP-HA-DAT Immunoblot Bands. First lane: molecular weight ladder. Second lane: YFP-HA-DAT with two bands representing mature DAT (110 kDa) and immature, ER-resident DAT (75 kDa). [file Image_1.TIF]

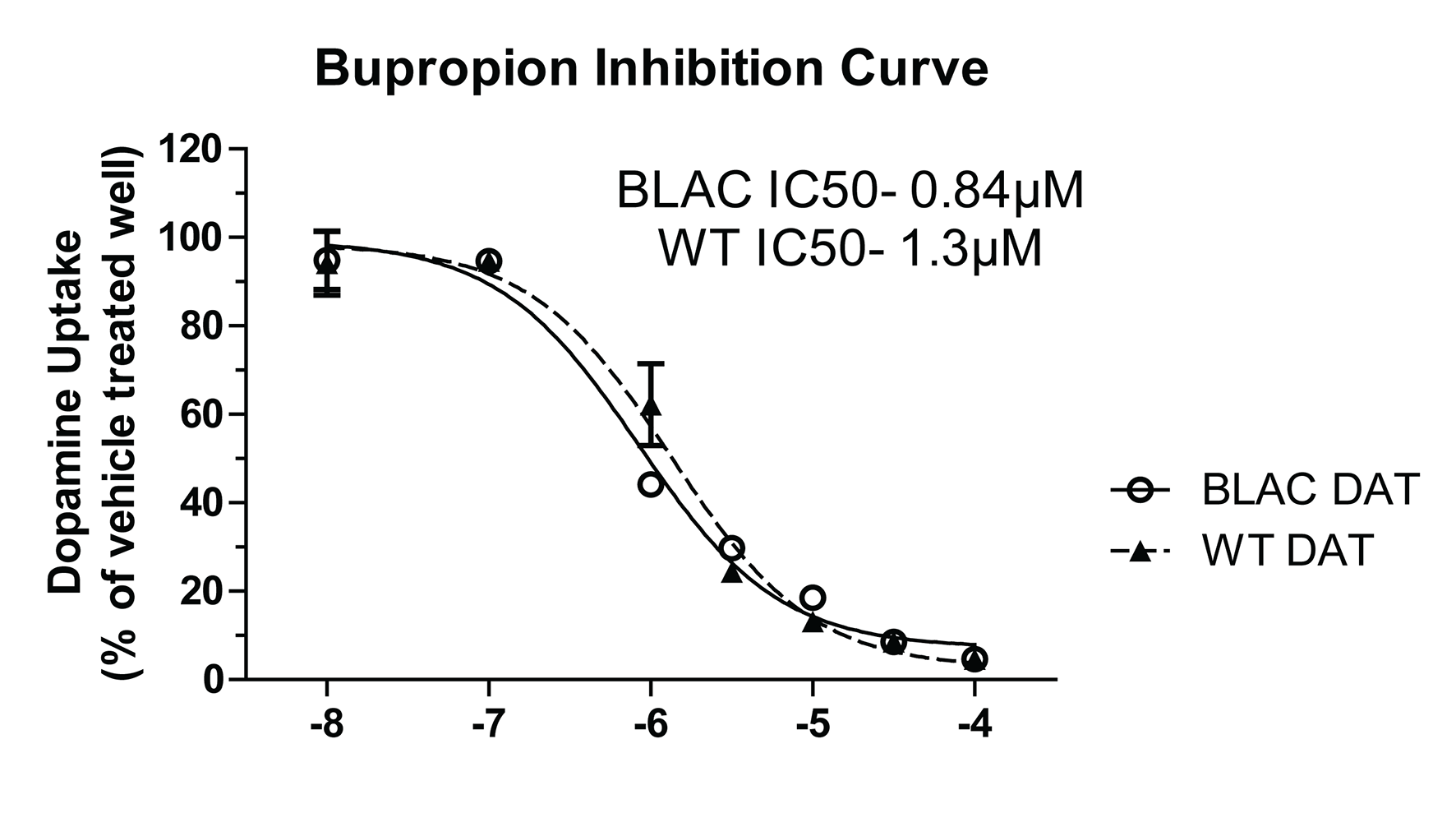

Supplement: Supplementary Figure 2 — Dose response inhibition of dopamine uptake by bupropion on WT and YFP-HA-βlac-DAT. Data were normalized to vehicle treatment. Data are means ± S.E.M. [file Image_2.TIF]

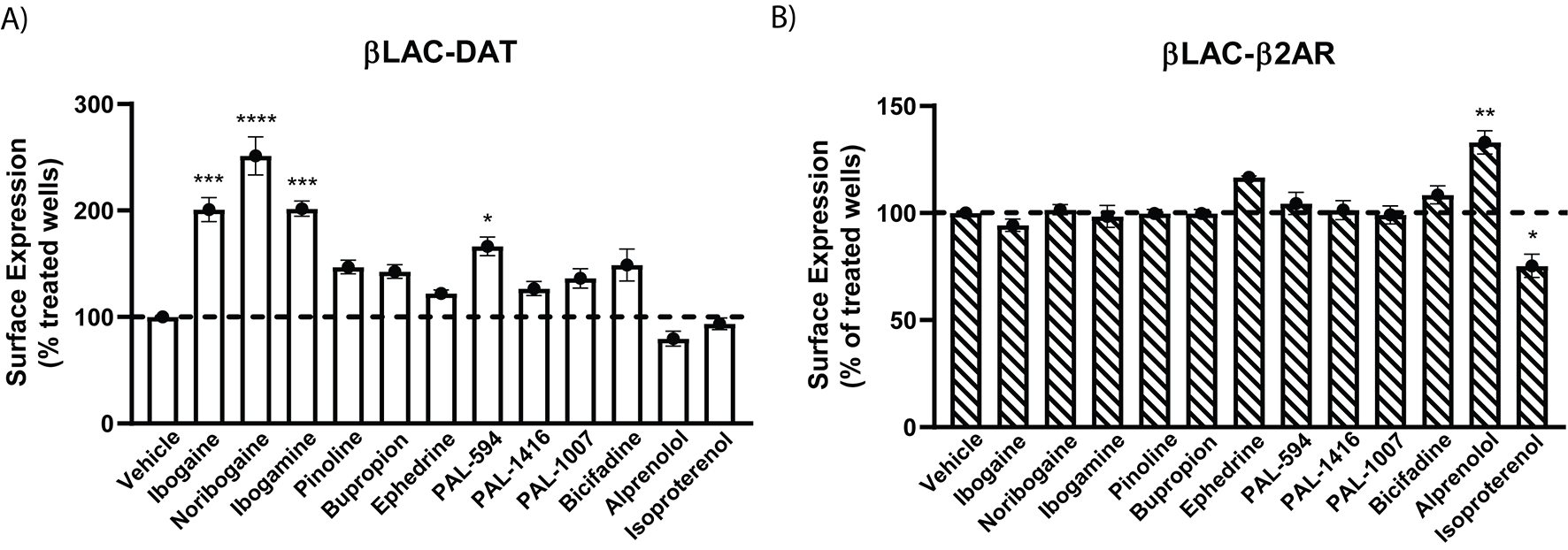

Supplement: Supplementary Figure 3 — EMax comparison between βlac-DAT and βlac-β2AR surface expression of hit compounds showing selectivity of chaperones for βlac-DAT. (A) Summary of the maximum observed effects of hit compounds on WT DAT surface expression. Data were normalized to vehicle treatment and analyzed with one-way ANOVA (****p < 0.0001, ***p < 0.001, **p < 0.01, and *p < 0.05, one-way ANOVA compared to vehicle with Dunnett’s test). (B) Summary of the maximum observed effects of hit compounds on β2AR. Data were normalized to vehicle treatment and analyzed with one-way ANOVA (****p < 0.0001, ***p < 0.001, **p < 0.01, and *p < 0.05, one-way ANOVA compared to vehicle with Dunnett’s test). The final concentration of drugs used was 100 μM, except for ephedrine at 1 mM. Data are means ± S.E.M; n = 3–6. [file Image_3.TIF]

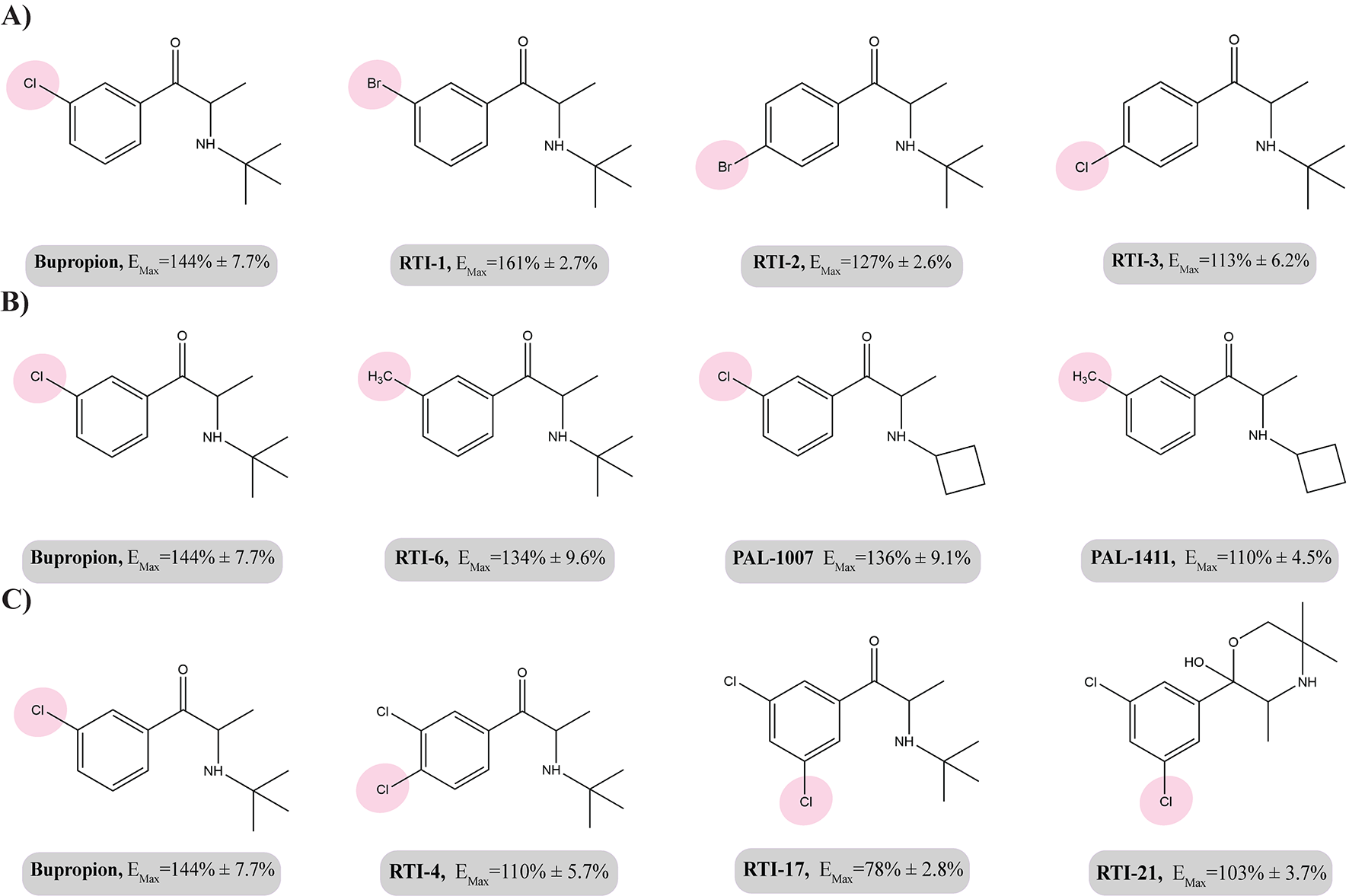

Supplement: Supplementary Figure 4 — Impact of phenyl substitutions on the efficacy of bupropion in the WT DAT blac assay. (A) Impact of para substitution compared to meta substitution on chaperone efficacy. (B) Impact of phenyl substitution at the meta position on chaperone efficacy. (C) Impact of addition of a second Cl on chaperone efficacy. [file Image_4.TIF]

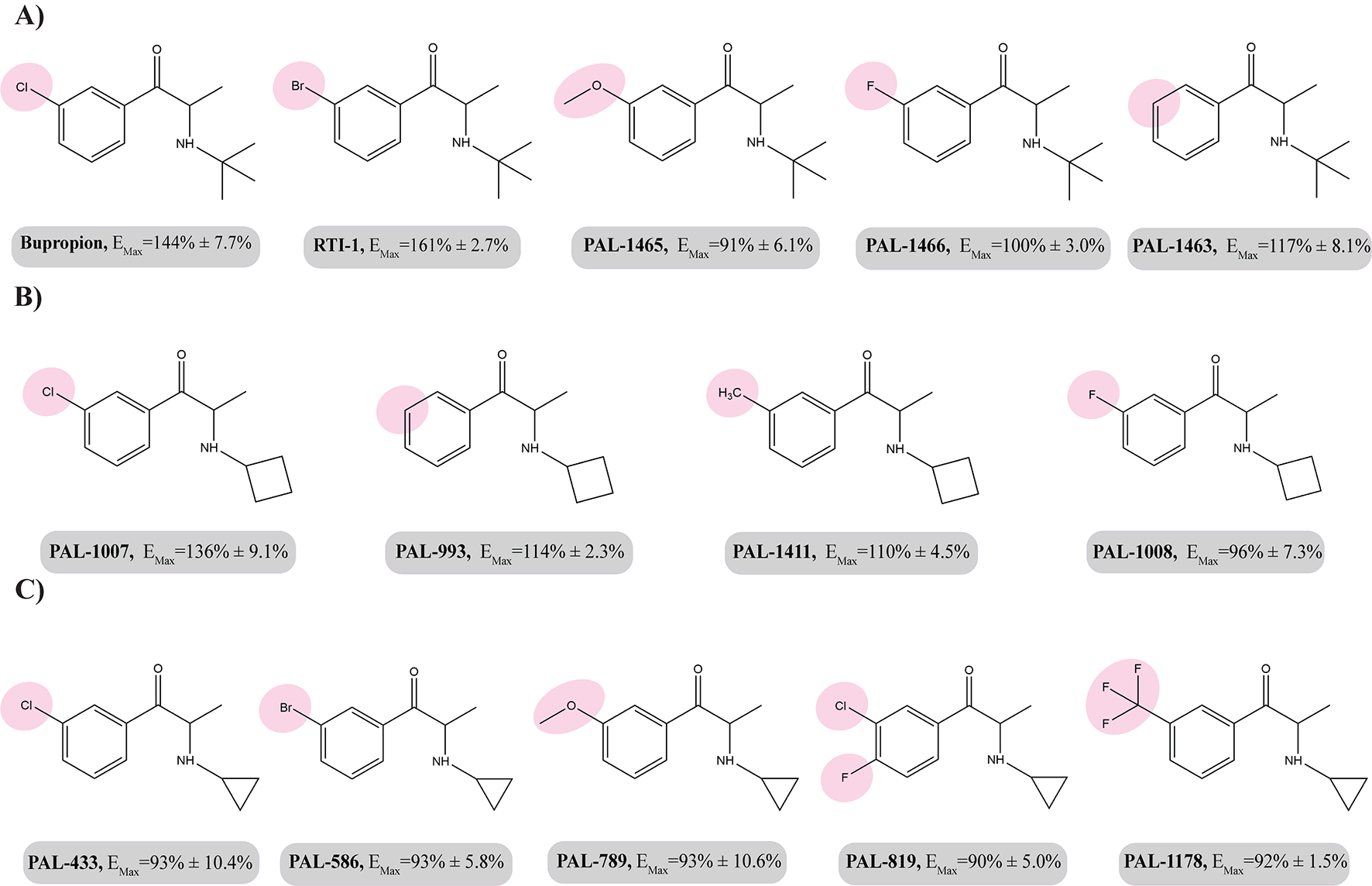

Supplement: Supplementary Figure 5 — Broader study of the impact of phenyl substitutions on the efficacy of bupropion in the WT DAT blac assay. (A) Impact of 3-phenyl substitutions on chaperone efficacy of compounds with an N-tertbutyl group. (B) Impact of 3-phenyl substitutions on chaperone efficacy of compounds with an N-cyclobutyl group. (C) Impact of 3-phenyl substitutions on chaperone efficacy of compounds with an N-cyclopropyl group. [file Image_5.TIF]

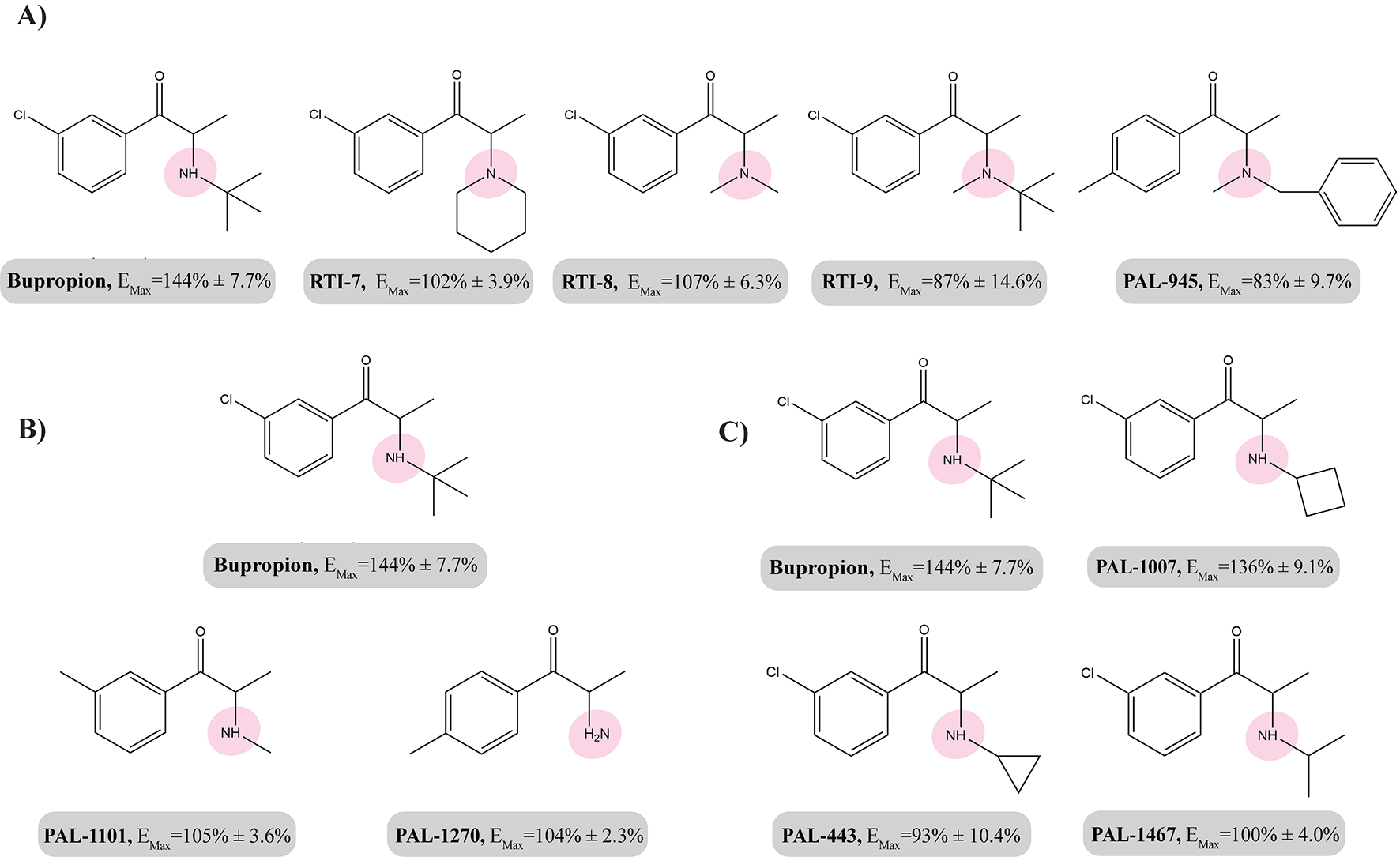

Supplement: Supplementary Figure 6 — Impact of N-alkylation on the efficacy of bupropion in the WT DAT blac assay. (A) Impact of tertiary amines on chaperone efficacy. (B) Impact of smaller alkyl groups on chaperone efficacy. (C) Impact of iso-alkyl groups on chaperone efficacy. [file Image_6.TIF]

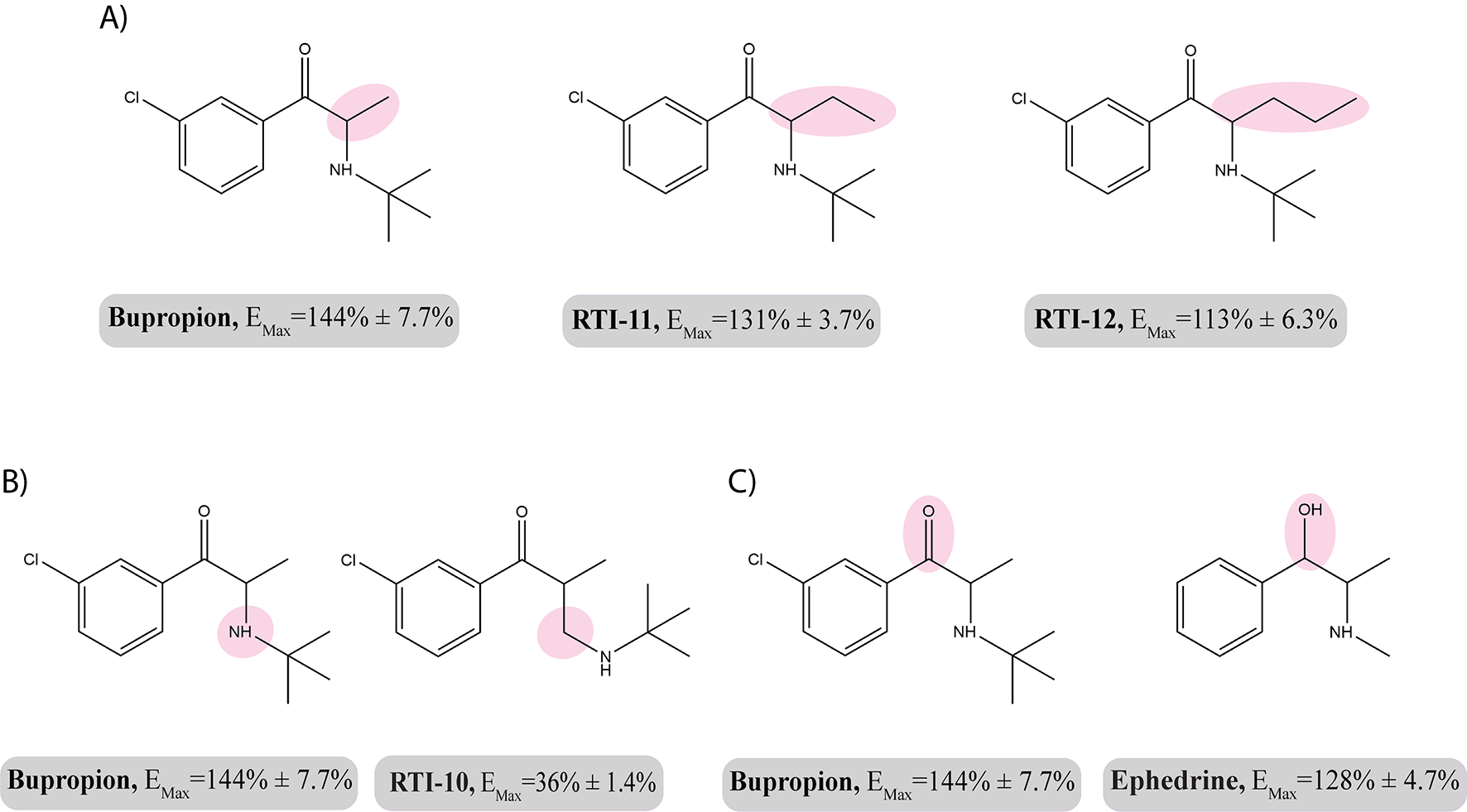

Supplement: Supplementary Figure 7 — Effect of the alkyl chain length and ketone substituent on the efficacy of bupropion in the WT DAT blac assay. (A) Impact of alkyl chain length on chaperone efficacy. (B) Impact of the addition of a methylene group on chaperone efficacy. (C) Impact of the addition of a ketone on chaperone efficacy. [file Image_7.TIF]

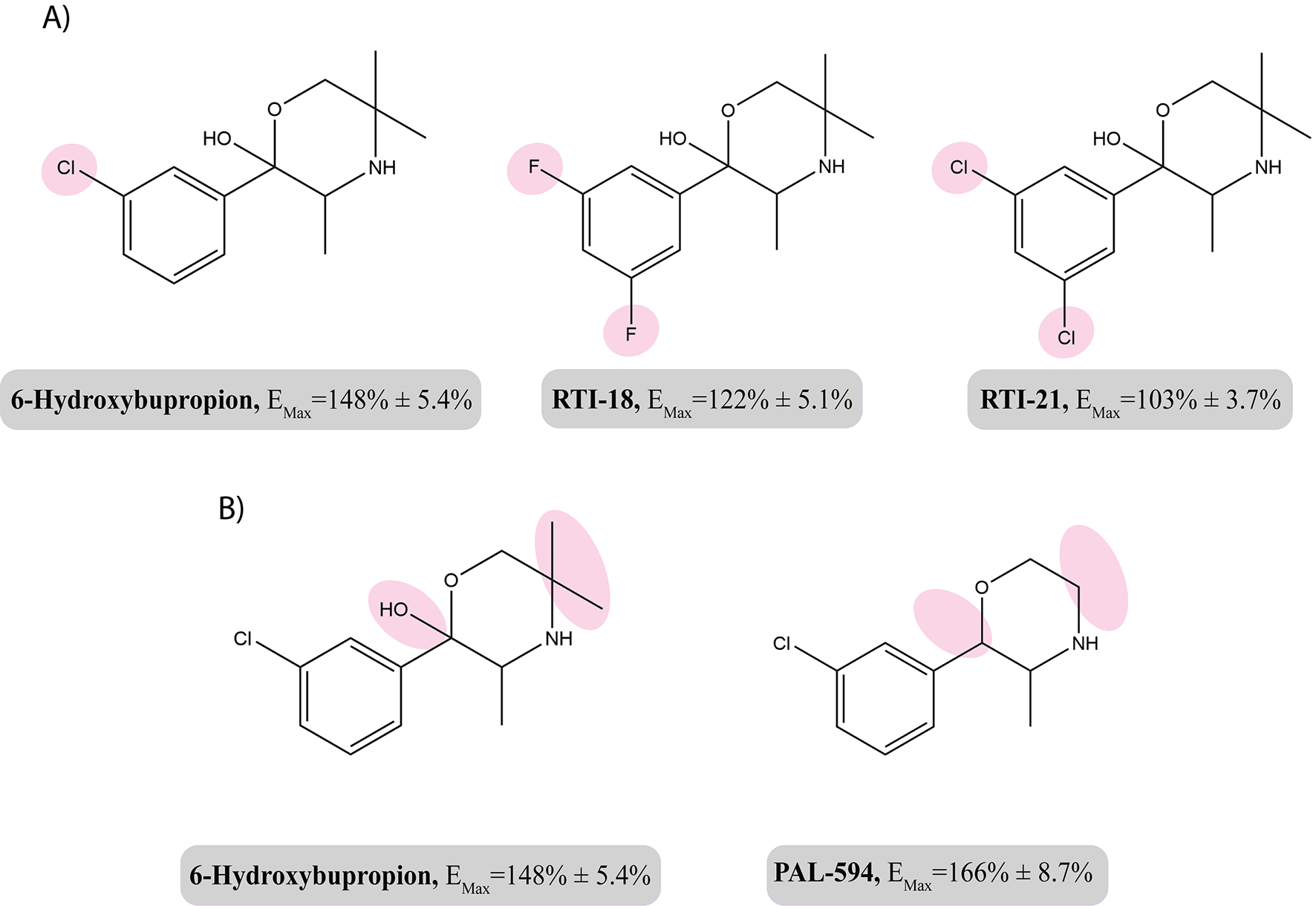

Supplement: Supplementary Figure 8 — Efficacy of 6-hydroxybupropion in the WT DAT blac assay.(A) Impact of the addition of a second halogen on chaperone efficacy of 6-hydroxybupropion. (B) Impact of the removal of hydroxyl and methyl groups from 6-hydroxybupropion on chaperone efficacy. [file Image_8.TIF]

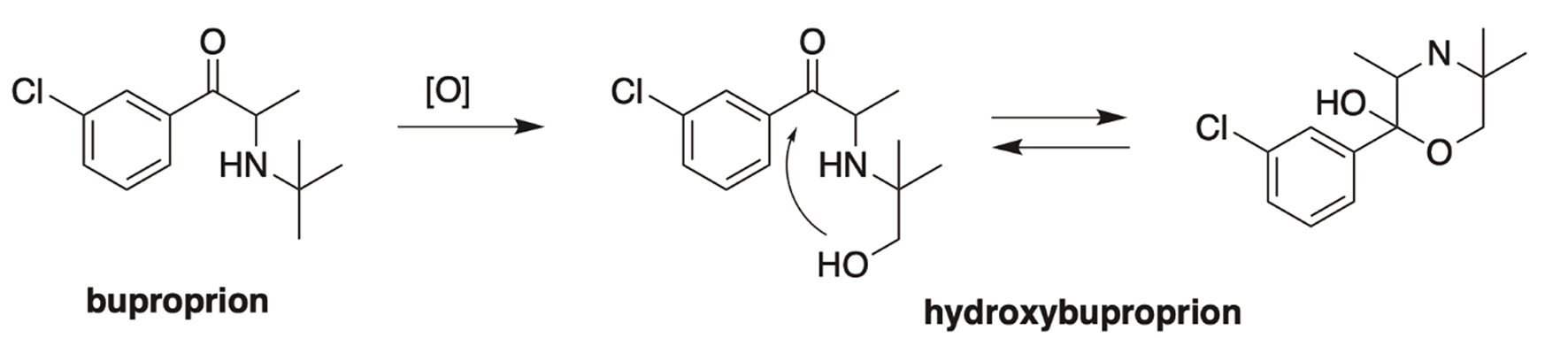

Supplement: Supplementary Figure 9 — Demonstration of bupropion converted to its metabolite 6-hydroxybupropion. [file Image_9.TIF]

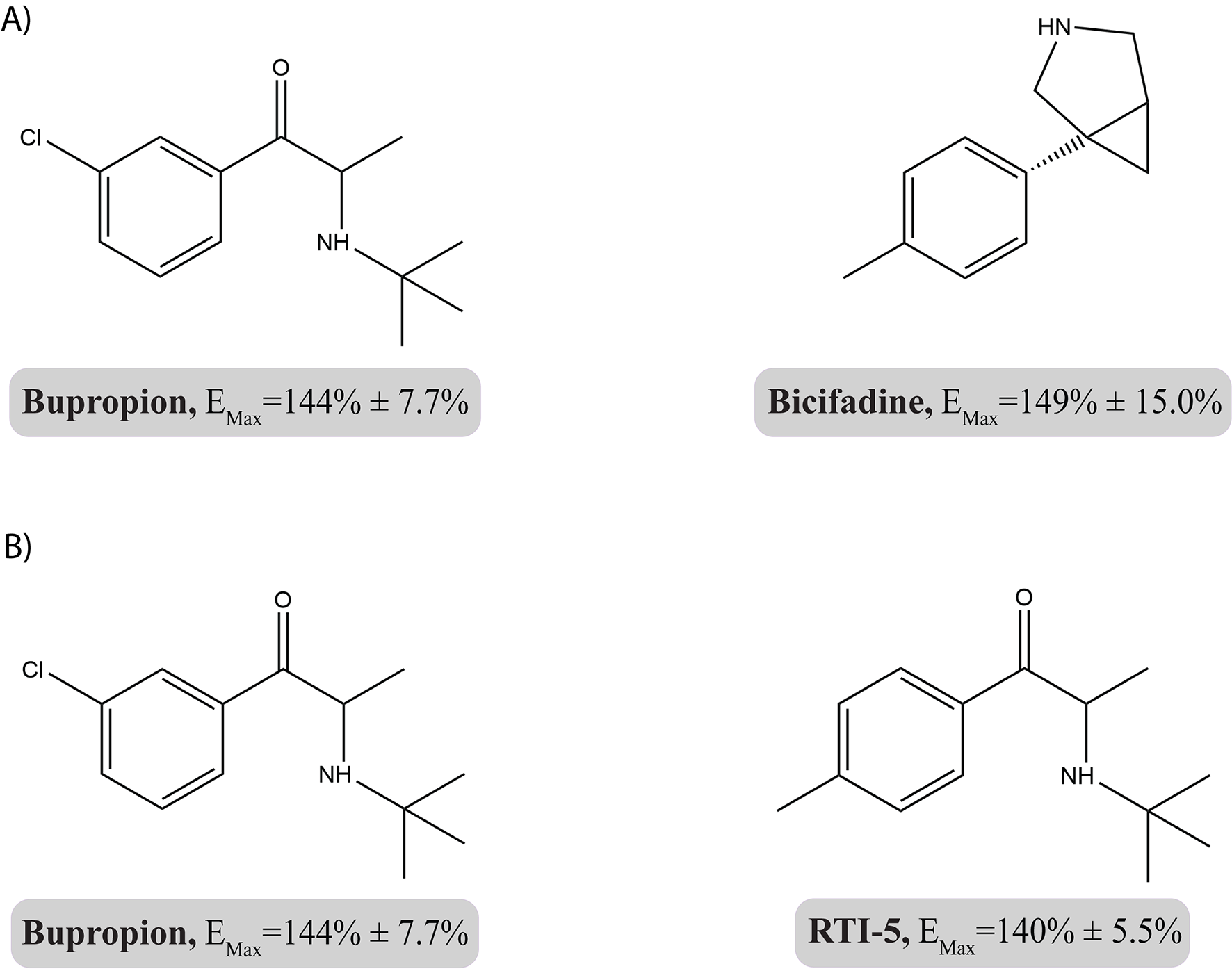

Supplement: Supplementary Figure 10 — Bicifadine and RTI-5 are potential bupropion analogues to be explored further for SAR analysis. (A) Bupropion and bicifadine have similar chaperone efficacy. (B) Bupropion and RTI-5 have similar chaperone efficacy. [file Image_10.TIF]

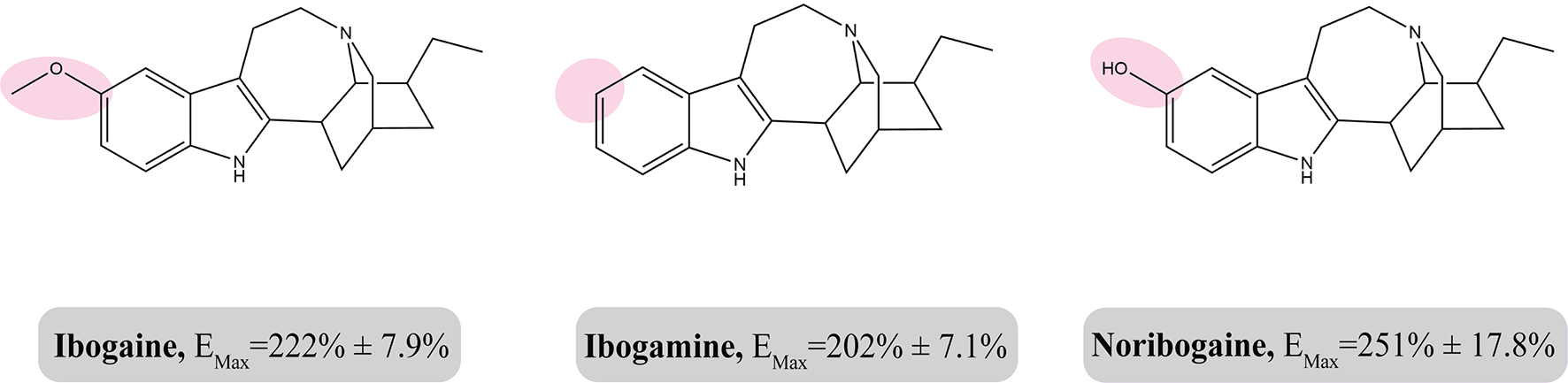

Supplement: Supplementary Figure 11 — Effect of the hydroxyl substitution on the efficacy of ibogaine in the WT DAT βlac assay. [file Image_11.TIF]

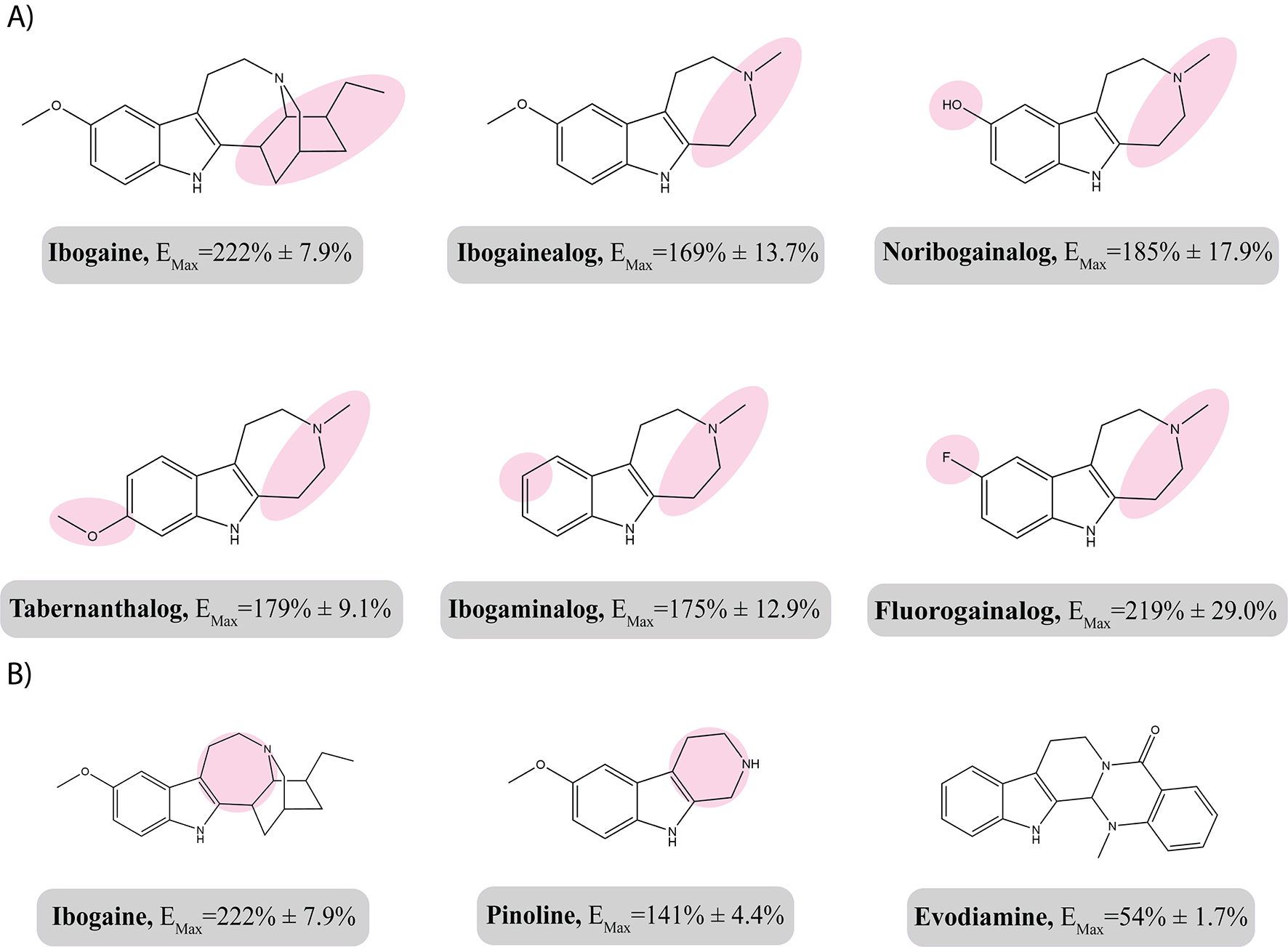

Supplement: Supplementary Figure 12 — Effect of the isoquinuclidine substituent and ring size on the efficacy of ibogaine in the WT DAT blac assay. (A) Impact of the removal of the isoquinuclidine substituent in addition to various phenyl substitutions on chaperone efficacy. (B) Impact of ring size on chaperone efficacy. [file Image_12.TIF]

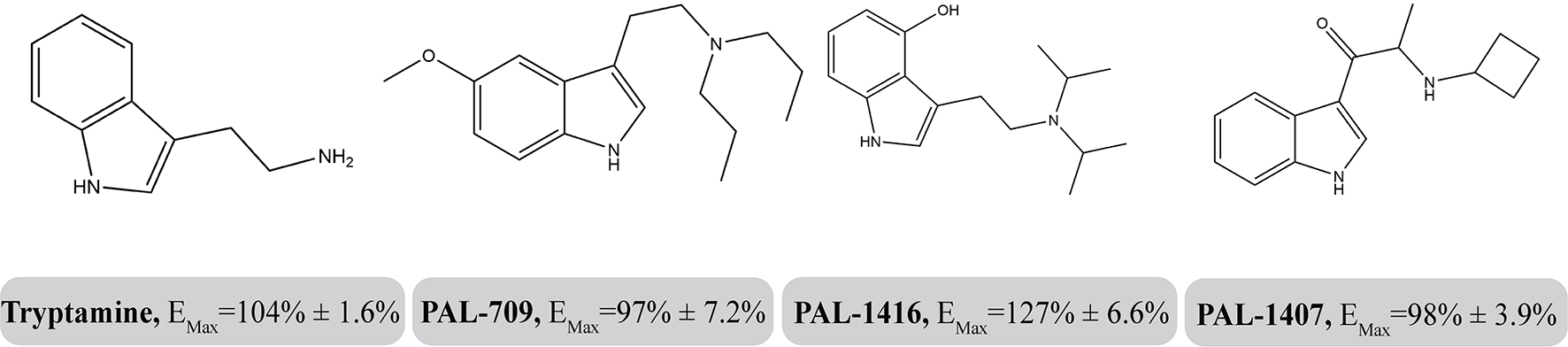

Supplement: Supplementary Figure 13 — Effect of the tryptamines on the efficacy of tryptamine analogs in the WT DAT βlac assay. [file Image_13.TIF]

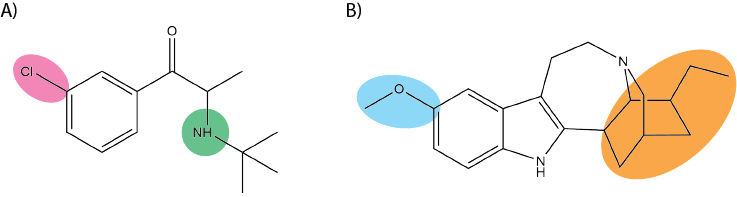

Supplement: Supplementary Figure 14 — Key structural features of bupropion and ibogaine for pharmacological chaperone efficaciousness. (A) Bupropion’s key structural features for pharmacological chaperone activity include the halogen derivation at the meta position (pink) and the secondary amine (green). (B) Ibogaine’s key structural features for pharmacological chaperone activity include hydroxyl substitution (blue) and the presence of the isoquinuclidine group (orange). [file Image_14.PNG]
